# Over 100-Year Preservation and Temporal Fluctuations of Cell Wall Polysaccharides in Marine Sediments

**DOI:** 10.3389/fpls.2022.785902

**Published:** 2022-04-18

**Authors:** Armando A. Salmeán, William George Tycho Willats, Sofia Ribeiro, Thorbjørn Joest Andersen, Marianne Ellegaard

**Affiliations:** ^1^Department of Plant and Environmental Sciences, University of Copenhagen, Frederiksberg, Denmark; ^2^Department of Technology, University College Copenhagen, Copenhagen, Denmark; ^3^School of Natural and Environmental Sciences, Newcastle University, Newcastle upon Tyne, United Kingdom; ^4^Geological Survey of Denmark and Greenland, Copenhagen, Denmark; ^5^Department of Geosciences and Natural Resource Management, University of Copenhagen, Copenhagen, Denmark

**Keywords:** comprehensive microarray polymer profiling (CoMPP), immunolabeling, geochemical proxy, Koljö Fjord, North Atlantic Oscillation (NAO index)

## Abstract

Polysaccharides constitute an important carbon pool in marine systems, but much is still unknown about the fate and degradation of these compounds. They are derived partly from production *in situ*, and in coastal areas, they are partly terrestrially derived, originating from freshwater runoff from land. The aim of this study was to test the applicability of high-throughput polysaccharide profiling for plant and algal cell-wall compounds in dated sediment cores from a coastal marine environment, to examine the preservation of cell-wall polysaccharides and explore their potential as proxies for temporal environmental changes. Preserved compounds and remains of organisms are routinely used as paleoenvironmental proxies as the amount and composition of different compounds that can provide insight into past environmental conditions, and novel means for reporting environmental changes are highly sought.

## Highlights

- A total of 30 different polysaccharide epitopes were detected in marine sediment samples by probing with plant and brown algal antibodies and carbohydrate-binding module (CBM) probes, using comprehensive microarray polymer profiling (CoMPP).
- Most of these epitopes were preserved at least 100 years, and some to the bottom of the sediment core (approximately 200 years), notably those detected by antibodies and CBM to fucose-containing sulfated polysaccharides (FCSP), cellulose, and the hemicelluloses xylan, xyloglucan, and mixed-linkage (1→3)(1→4)-β-D-glucan (MLG).
- Profiles of epitopes varied over time, with indications of links to environmental variability.
- The potential to use this methodology to identify novel geochemical proxies of environmental change is discussed.

## Introduction

The aim of this study was to test high-throughput polysaccharide profiling of plant and algal cell-wall compounds in marine sediments. We wished to determine the temporal preservation of polysaccharide epitopes and further explore the potential of these compounds as proxies for temporal environmental changes in a coastal marine setting.

Dissolved organic carbon (DOC) is by far the largest planetary pool of organic carbon (Kaiser, 2011). The photosynthetic production of marine microalgae, which is estimated to comprise half the global primary production (Field et al., 1998), contributes to this. In marine coastal areas, terrestrially derived material can also constitute an important part of the carbon pool. Less is known about the fate of terrestrial organic matter in the ocean (Hedges et al., 1997; Cragg et al., 2020) although understanding this is central for Earth System Models (Gontikaki et al., 2015). Degradation and processing of marine algal polysaccharides are also poorly understood. As organisms die, unless degraded in surface layers, they sink to the seafloor and either become degraded or preserved in sediment layers. Algal polysaccharides are important components of this process (Youssef et al., 2014 and references therein). These processes form a part of the biological pump, sequestering carbon to sea sediment, and if buried, or in the deep sea, taking it out of the active global carbon cycle.

Koljö Fjord is a semi-enclosed sill fjord with limited water exchange in the Kattegat/Skagerrak. The fjord is, therefore, strongly affected by runoff from land and exhibits brackish conditions and a stratified water column with 1–2 m of fresher water at the surface (Nordberg et al., 2001). The Bäveån river, with a catchment of ca. 300 km^2^, is the main point source of freshwater to the fjord. The strong stratification in the fjord leads to a more or less stagnant bottom water, with periodically occurring hypoxic or anoxic conditions (Gustafsson and Nordberg, 1999). In such environments, with decreased oxygen exposure times, a strong case can be made for paleo reconstruction of past organic matter composition sources (Bianchi and Canuel, 2011), as such sediments are often both undisturbed by animal activity (bioturbation), and remains of organisms from the water column are often well-preserved, due to limited bacterial activity. The lower part of Bäveån (closest to Koljö Fjord) is a nature reserve dominated by conifers on higher land and deciduous forest, grasses, and wetlands in the lower areas (information from Uddevalla municipality).

Studies of macroalgae in fjords and embayments near Koljö Fjord show that the shallow areas are characterized by diverse flora, with the highest area coverage, as well as the highest species diversity within the Rhodophyceae (red algae) and Phaeophyceae (brown algae) (Eriksson et al., 2002). Eriksson et al. (2002) also found that the depth-coverage had been significantly reduced between 1941 and 1998, most pronounced below ca. 3 m water depth and that small ephemeral and filamentous macroalgae had increased in relative abundance. Other studies have also seen an increase in green filamentous algae over the past decades (Cossellu and Nordberg, 2010).

Remains of different organisms in such undisturbed sediments can constitute time series of the biological and chemical conditions of a water body, and sediment proxies of the environment can broadly speaking be physical remains of different organisms and molecules from either the organisms or the water itself. Such geochemical proxies can be organic or can constitute isotopes, isotope ratios, or heavy metals. Organic geochemical proxies are molecules, produced by living organisms in the past that are preserved in marine and/or freshwater sediments. Central requirements for a geochemical proxy are that it is well-preserved in aquatic sediments and that it shows patterns of response to environmental forcing. Photosynthetic pigments (Reuss et al., 2005; Ellegaard et al., 2006), sterols, alkenones, and other lipids (Smittenberg et al., 2004) have most commonly been used as geochemical proxies of paleoenvironmental conditions. Due to the dominance of carbohydrates in organic matter in aquatic systems, there is considerable interest in their application as biomarkers (Bianchi and Canuel, 2011). The monosaccharide composition (ratio of hexoses to pentoses) in sediments has long been used as a proxy for the origin of the carbohydrates in soils and sediments (microbial or plant-derived, Spohn and Giani, 2012). Sugars have been used as paleoenvironmental indicators of the source of plant material in peat bogs (Jia et al., 2008), and monosaccharide ratios have been used to distinguish between terrestrial and marine carbohydrate sources in seawater (Cowie and Hedges, 1984). However, polysaccharides have more rarely been reported as paleoenvironmental proxies.

Koljö Fjord has been the target of several paleoenvironmental studies, and patterns in environmental change derived from studies of temporal changes in dinoflagellate cyst and diatom communities were used to compare with the temporal patterns in the polysaccharides.

Harland et al. (2004) studied dinoflagellate cysts in Koljö Fjord sediment layers dating back to pre-1855 and found large changes in the dinoflagellate cyst community ca. 1940 and pre-1855. McQuoid and Nordberg (2003) studied diatoms in Koljö Fjord sediment layers dating back to pre-1840 and similar changes ca. 1930 and pre-1840. Both studies linked these changes to shifts in environmental conditions, primarily shifts in the pattern of the North Atlantic Oscillation (NAO). The NAO is a major pattern of atmospheric variability in the Northern hemisphere (van Loon and Rogers, 1978), and the winter NAO index influences precipitation and sea-surface temperatures (Schimanke et al., 2012).

Comprehensive microarray polymer profiling (CoMPP) is a high throughput technique, which allows the analysis of multiple samples with the advantage of not needing complex separation techniques such as chromatography or hydrolysis. Samples undergo a fractionation with different solvents and later are probed using specific monoclonal antibodies and carbohydrate-binding module (CBM) probes. These probes bind to target epitopes in the sample matrix. Revealing the positive reaction requires an enzymatic process, which generates a signal (fluorescent or chemometric) on the array surface that can be interpreted with a dedicated software (Moller et al., 2007). This semi-quantitative method has been largely used with success for multiple types of polysaccharides in different matrixes, among them from plant and algal cell-wall plants (Sørensen et al., 2008; Salmeán et al., 2017a) but also marine animals (Salmeán et al., 2017b).

The target site is, thus, known to present a good environment for the preservation of biological and geochemical remains and to be subject to documented environmental change over time. Our aim was, in this setting, to test the applicability of high-throughput polysaccharide profiling for detecting polysaccharides in sediment cores, with the final aim to explore the potential of such compounds as proxies for temporal environmental changes.

## Materials and Methods

### Sampling and Dating

**FIGURE 1:**
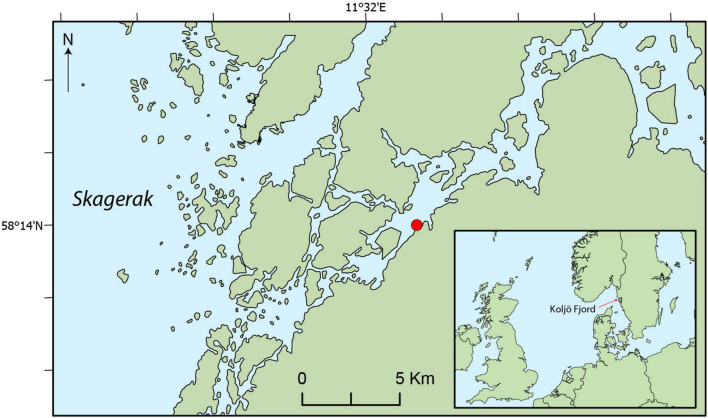
Map showing the Koljö Fjord sampling site on the west coast of Sweden.

**FIGURE 2:**
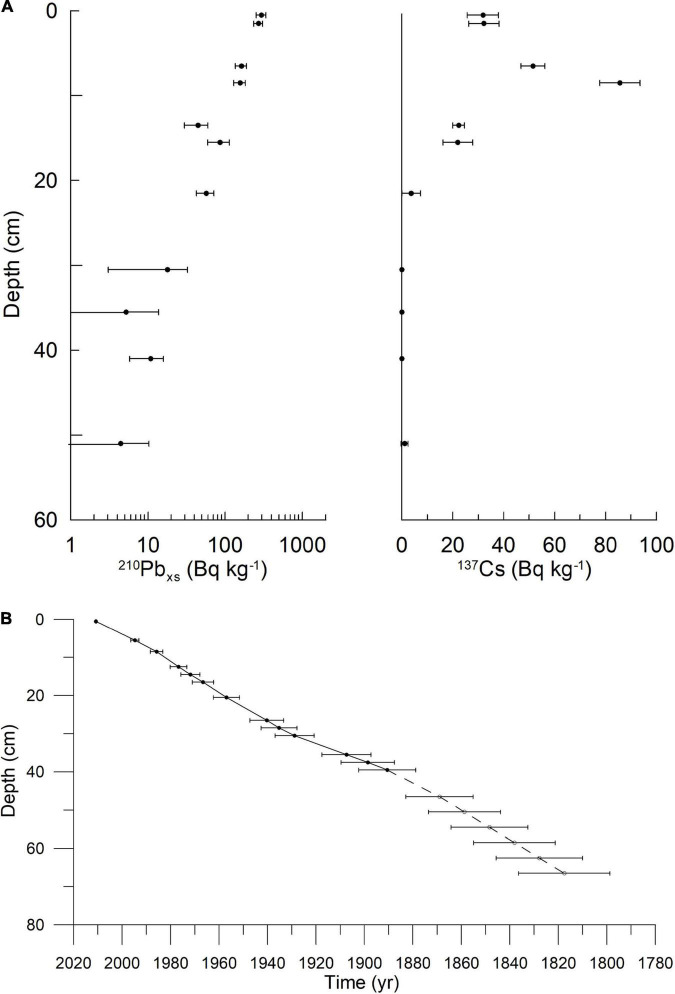
**(A)** Activity of ^210^Pb (left) and ^137^Cs (right) with depth. These data were used to create the age-depth model. **(B)** Age-depth model. Both for the core from Koljö Fjord KF12/5.

Surface, or near-surface, sediment from one marine sediment core from each of Mariager Fjord, Koljö Fjord, and Sermilik was used in the initial screening for polysaccharides. Sampling information and coordinates for these three sites are given in Table 1. The sample from Koljö Fjord, Sweden (Figure 1) had the largest diversity and relative amounts of polysaccharides and was, therefore, chosen for further analysis, using a 67-cm long sediment core (KF12/5, refer to Table 1). The core was x-rayed before subsampling at 1-cm resolution [refer to Ribeiro et al. (2011) for details of the subsampling procedure]. Samples were stored in the dark at 4°C until further processing. Eighteen samples from KF12/5, distributed approximately evenly along the core KF12/5 (2–4 cm apart), were used for further analysis. The core chronology in the core was established by means of ^210^Pb dating with ^137^Cs-peaks for verification (data shown in Figure 2A). The ^210^Pb-based chronology was calculated using a modified CRS-model (Andersen, 2017) in which the activity below 41 cm was calculated using a regression of activity of unsupported ^210^Pb vs. cumulative mass depth.

**TABLE 1:** Coordinates for the three sampling sites in the pilot study and methods used for coring.

Location	Coordinates	Core code	Sampling date	Corer
Koljö Fjord, Sweden	58°13.591 N	KF12/5	March 13, 2012	Rumohr lot
	11°34.293 E			
Mariager Fjord, Denmark	56°39.814 N	MF13/2	September 24, 2013	Supercorer
	9°58.517 E			
Sermilik Fjord, East Greenland	66°5.149 N	S11	August 2012	Rumohr lot
	37°45.728 W			

*Core KF12/5 was selected for the main study.*

### Sediment Extractions

The first batch (the three surface sediment samples) constituted the pilot project and was used to test the methodology. The three samples were homogenized and freeze-dried. The first stage was the preparation of alcohol insoluble residue (AIR), which serves to concentrate larger polymers and remove water. To prepare AIR, 1 g of freeze-dried material was suspended in falcon tubes to a final volume of 5 ml of 70% ethanol, stirred for 3 min, and centrifuged at 40,000 rpm for 10 min. The resulting pellet was resuspended to a final volume of 5 ml 96% ethanol, and the previous step was repeated two times more with 1:1 methanol:chloroform and 100% acetone. All the resulting supernatants were kept and freeze-dried for analysis, and the final pellet was also freeze-dried.

Since the sediments are marine, a sequential chemical fractionation with the aim to extract FCSP and alginates (Salmeán et al., 2017b), but also pectins, hemicelluloses, and cellulose, was performed as follows: 1 g of freeze-dried material was dissolved in up to 3 ml of water at 80°C with stirring in a tissue lyser (1 h) and separated by centrifugation as above. The pellet was later dissolved in up to 0.2 M HCl for 1 h with stirring and separated. Finally, the pellet was dissolved in up to 3 ml of 4 M NaOH and similarly extracted for 1 h. The final pellet was kept for future analysis, while all the supernatants were freeze-dried.

Since the results from the pilot project provided more land plant signals than originally expected, for the core KF12/5, CDTA was used instead of HCl to extract more pectins.

### Comprehensive Microarray Polymer Profiling—Antibodies, Printing, and Probing

The supernatants from AIR and the subsequent chemical fractionations were freeze-dried and redissolved up to a 1-ml Arrayjet buffer (55.2% glycerol, 44% water, and 0.8% Triton X-100) and were printed on to nitrocellulose as previously described (Salmeán et al., 2018). The resulting microarrays were probed as described in the study by Salmeán et al. (2018) using the following monoclonal antibody and CBM probes diluted in phosphate-buffered saline (PBS) with 5% milk (Table 2): anti-His tagged CBM3a at a concentration of 10 μg/ml, CBM30 at 5 μg/ml, anti-mouse INRA-RU1 and INRA-RU2, and BS-400-4, BS-400-2, and BS-400-3 at a concentration of 1/10, and the following anti-rat antibodies (all of them at a concentration of 1/10): JIM5, JIM7, LM19, LM20, LM5, LM6, LM10, LM11, LM21, LM15, JIM8, JIM20, and LM7.

**TABLE 2:** Specificities of the probes used in this study, organized in alphabetical order.

Probe	Recognised epitope structure	References
BAM-1	Un-sulfated epitope present in sulfated fucan	Torode et al., 2015
BAM-2	Sulfated epitope present in sulfated fucan	Torode et al., 2015
BAM-3	Possibly sulfated epitope present in sulfated fucan	Torode et al., 2015
BS-400-2	Callose, (1→3)-β-D-glucan	Meikle et al., 1991
BS-400-3	(1→3)(1→4)-β-D-glucan	Meikle et al., 1994
BS-400-4	Mannan(1→4)-β-D-mannan	Pettolino et al., 2001
CBM3a	Crystalline cellulose/XG	Blake et al., 2006; Hernandez-Gomez et al., 2015
CBM6	Amorphous cellulose, β-1,4-xylan,β-1,3-glucan, (1→3)(1→4)-β-D-glucan, β-1,4-glucan	Fernandes et al., 1999; Czjzek et al., 2001; Boraston et al., 2003; Henshaw et al., 2004; Pires et al., 2004
CBM30	HE cellulose/MLG/XG	Najmudin et al., 2006
INRA-RU1	Rhamnogalacturonan I	Ralet et al., 2010
INRA-RU2	Rhamnogalacturonan I	Ralet et al., 2010
JIM5	Homogalacturonan with a low DE	Clausen et al., 2003
JIM7	Homogalacturonan with a high DE	Willats et al., 2001; Clausen et al., 2003
JIM8	AGP	McCabe et al., 1997
JIM13	AGP	Knox et al., 1991
JIM16	AGP	Knox et al., 1991
JIM20	Extensin	Smallwood et al., 1994
LM5	Pectinside chains RGI, (1→4)-β-D-galactan	Jones et al., 1997
LM6	Side chains RGI, (1→5)-α-L-arabinan	Willats et al., 1998; Lee et al., 2005; Verhertbruggen et al., 2009
LM7	Homogalactauronan with an intermediate DE, non-blockwise distribution of MeOH/Alginate	Willats et al., 2001; Torode et al., 2015
LM8	Xylogalacturonan	Willats et al., 2004
LM10	Xylan, (1→4)-β-D-xylan	McCartney et al., 2005
LM11	Xylan, (1→4)-β-D-xylan/arabinoxylan	McCartney et al., 2005
LM12	Feruloylate on any polymer	Pedersen et al., 2012
LM13	Linearised (1→5)-α-L-arabinan	Moller et al., 2008; Marcus et al., 2010
LM14	AGP	Moller et al., 2008; Pedersen et al., 2012
LM15	Xyloglucan (XXXG motif)	Marcus et al., 2008
LM16	homogalactauronan, 6′-β-D-galactosyl-β-(1→4)-D-galactotriose [Processed (1→5)-α-L-arabinan]	Verhertbruggen et al., 2009
LM18	Partially methylesterified homogalacturonan	Verhertbruggen et al., 2009
LM19	Partially methylesterified homogalacturonan	Verhertbruggen et al., 2009
LM20	Partially methylesterified homogalacturonan	Verhertbruggen et al., 2009
LM21	Mannan, (1→4)-β-D-mannan/galactomannan/glucomannan	Marcus et al., 2010
LM25	Xyloglucan	Pedersen et al., 2012
MAC207	AGP, β-linked GlcA	Pennell et al., 1989

Samples from core KF12/5 were printed as described above and probed against the same anti-mouse antibodies under the same conditions except for INRA RU1 and INRA RU2, which were excluded this time, the same anti-His antibodies also including CBM6 (at a concentration 10 μg/ml), and all the anti-rat antibodies cited before, including the following as well (Table 2): LM18, LM16, LM8, LM5, LM6, LM13, JIM13, JIM16, LM14, LM12, JIM20, LM7, MAC207, BAM1, BAM2, and BAM3, at a concentration of 1/10.

The arrays were scanned, and their signals quantified as described by Hervé et al. (2016) to obtain heat maps.

## Results

Like previous cores taken at the same site in Koljö Fjord (Ribeiro et al., 2011), the age model for the core is robust, with relatively low errors on the age in the top part of the core, slightly higher (ca. 10–30-year span pr. cm slice) in the bottom part of the core (Figure 2B). The 67 cm of the core corresponds to ca. 200 years of sedimentation (Figure 2B). The pilot study showed that antibodies developed mainly for epitopes of polysaccharides in higher land plants (McCartney et al., 2005) were able to detect epitopes in carbohydrates extracted from marine sediment samples (Table 3) and those extraction methods previously employed for marine materials (Salmeán et al., 2017a,b) also appeared to be relevant for this type of material. Thus, signals were detected in the surface samples from 14 out of 21 epitopes, and all three sequential extractions eluted different amounts and/or types of materials. Therefore, we selected the site with the surface sample with the most hits (which was Koljö fjord) and continued our study on a core from this site.

**TABLE 3:** Heat map for the surface sediment samples for different extractions (pilot project, sequentially extracted in this order: water, HCl, and NaOH).

	Site	CBM3a	CBM30	JIM5	JIM7	LM19	LM20	INRA-RU1	INRA-RU2	LM5	LM6	LM10	LM11	BS-400-4	LM21	LM15	LM25	BS-400-2	BS-400-3	JIM8	JIM20	LM7
Water	MF	8	0	0	0	0	0	0	0	0	0	0	0	10	0	0	0	13	0	0	0	0
	KF	0	0	0	0	0	0	0	0	8	0	0	0	8	6	0	0	0	0	0	0	0
	S	0	0	0	0	0	0	0	0	0	0	0	6	0	0	0	0	6	7	0	0	0
HCl	MF	7	0	5	0	10	5	0	0	9	0	0	0	15	0	0	0	0	5	0	0	0
	KF	6	0	0	13	100	0	0	0	0	0	0	9	27	0	0	0	6	0	0	0	0
	S	0	0	0	0	0	0	0	0	0	0	0	13	0	0	0	0	0	0	0	0	0
NaoH	MF	26	22	0	0	0	0	0	0	0	0	0	0	11	7	0	0	30	12	0	0	0
	KF	22	24	0	0	0	0	0	0	0	0	0	7	17	16	0	6	14	13	0	0	0
	S	17	13	0	0	0	0	0	0	0	0	0	6	7	0	0	0	0	10	0	0	0

*The strength of the color is proportionate to the strength of the signal; the strongest signal is given the value 100 and the others valued relative to this signal. Epitopes with a relative signal lower than 5 in any of the samples were excluded.*

In the extractions from the core, KF12/5 signals were detected in all 18 samples by 30 out of 31 antibodies (Table 4). For some antibodies (i.e., LM20, LM13, and CMB6) there were, however, only few weak signals (Table 4). The strongest signals were seen in the antibodies raised against FCSP and hemicelluloses (refer to the discussion for interpretations of this). In the three samples below 56 cm (older than ca. 1850), there were fewer hits (5–10) in the heat maps, while above this depth there were, with a few exceptions (6 cm, 15 cm), more than 18 hits in each sample. A few antibodies and CBMs (i.e., CBM30, BS-400-3, BAM1, and BAM2) had hits in all samples.

**TABLE 4:** Heat map for NaOH extractions from the core samples from KF12/5.

		Cellulose			Pectin											Hemicellulose									AGP					FCSP				
					
Depth (cm)	Year (circa)	CBM3a	CBM30	JIM5–Homogalacturonan with a low DE	JIM7–Homogalacturonan with a high DE	LM7–Non-blockwise partially methyl-esterified HG/alginate	LM19–partially methylesterified homogalactauronan	LM20–Partially methylesterified homogalactauronan	LM18–Partially methylesterified homogalactauronan	LM16–Galactosyl residue(s) on rhamnogalacturonan I	LM8–Xylogalacturonan	LM5–(1→4)-β-D-galactan	LM6–(1→5)-α-L-arabinan	LM13–Linearised (1→5)-α-L-arabinan	LM10–(1→4)-β-D-xylan	LM11–(1→4)-β-D-xylan/arabinoxylan	BS-400-4–(1→4)-β-D-mannan	LM21–(1→4)-β-D-(galacto)(gluco)mannan	LM15–Xyloglucan (XXXG motif)	LM25–Xyloglucan	BS-400-2–(1→3)-β-D-glucan	BS-400-3–(1→3)(1→4)-β-D-glucan	CBM6–(1→3)(1→4)-β-D-glucan	JIM8	JIM13	JIM16	LM14	MAC207	BAM1–Un-sulfated epitope present in sulfated fucan	BAM2–sulfated epitope present in sulfated fucan	BAM3–Possibly sulfated epitope present in sulfated fucan	LM12–Feruloylate on any polymer	JIM20–Extensin
0–1	2011	0	19	6	7	0	12	0	7	0	0	9	5	0	10	10	29	11	7	27	50	65	0	0	6	6	15	9	63	39	0	6	8
5–6	1995	0	9	0	0	19	0	0	0	0	0	0	0	0	0	19	27	0	0	0	0	67	0	0	0	0	0	0	39	13	0	0	0
8–9	1986	0	24	14	12	0	16	0	10	6	8	12	11	5	14	19	39	18	11	41	27	55	5	6	9	7	13	10	58	50	0	8	7
12–13	1977	0	49	5	5	0	16	0	5	0	0	8	0	0	9	24	26	11	12	28	13	51	0	0	0	0	11	0	37	45	0	0	7
14–15	1972	0	32	0	0	0	7	0	0	0	0	0	0	0	0	6	10	0	0	0	0	52	0	0	0	0	6	0	32	24	0	0	0
16–17	1967	0	27	13	7	6	8	0	7	0	6	8	0	0	11	24	20	13	10	28	15	63	0	0	0	8	13	9	43	37	0	8	5
20–21	1957	10	38	8	0	0	0	5	9	0	0	6	0	0	0	13	16	8	9	23	16	70	0	0	7	0	19	8	45	42	0	0	0
26–27	1940	0	26	10	9	0	20	0	11	0	5	10	0	0	9	14	22	14	16	44	12	66	0	0	9	0	19	11	55	51	0	6	9
28–29	1935	22	34	13	10	5	12	0	10	6	8	12	7	0	10	19	32	15	12	49	26	56	0	7	6	8	18	12	51	58	0	7	8
30–31	1929	0	42	11	8	12	22	0	10	6	7	10	0	0	13	25	26	15	19	51	7	48	0	7	7	6	17	13	53	60	0	7	7
35–36	1907	0	41	13	12	0	17	0	9	6	8	12	7	0	10	27	22	14	11	47	8	60	5	9	6	7	19	13	61	60	0	11	8
37–38	1899	0	42	14	13	0	18	0	12	7	10	14	13	7	11	23	34	18	17	48	16	100	6	10	7	10	20	13	57	53	0	9	9
39–40	1891	17	29	12	8	0	9	0	11	0	6	10	5	0	8	14	12	13	15	44	7	73	0	6	11	7	24	12	56	55	0	8	7
46–48	1869	0	44	11	9	0	10	0	6	5	5	11	0	0	12	31	20	13	10	45	0	62	0	6	10	6	15	11	44	69	0	8	7
50–52	1859	0	26	7	8	0	7	0	7	0	0	9	0	0	7	17	18	11	7	33	0	66	0	0	5	5	13	8	45	60	0	5	0
54–56	1848	0	34	8	6	0	6	0	12	0	0	7	0	0	6	18	19	10	10	30	0	84	0	6	5	0	23	7	36	62	0	6	0
58–60	1838	9	14	0	0	0	0	0	11	0	0	0	0	0	0	0	11	0	0	5	13	52	0	0	0	0	5	0	15	45	0	0	0
62–64	1828	28	12	0	0	0	0	0	0	0	0	0	0	0	0	0	0	0	0	0	0	54	0	0	0	0	0	0	15	43	0	0	0
66–67	1818	0	14	0	0	0	0	0	0	0	0	0	0	0	0	0	10	0	0	0	0	57	0	0	0	0	0	0	15	40	0	0	0

*The strength of the color is proportionate to the strength of the signal; the strongest signal is given the value 100 and the others valued relative to this signal. Epitopes with a relative signal lower than 5 in any of the samples were excluded. CBM, carbohydrate-binding module; DE, degree of esterification; HG, homogalacturonan; AGP, arabinogalactan protein; FCSP, fucose-containing sulfated polysaccharides (fucoidans or fucans).*

**FIGURE 3:**
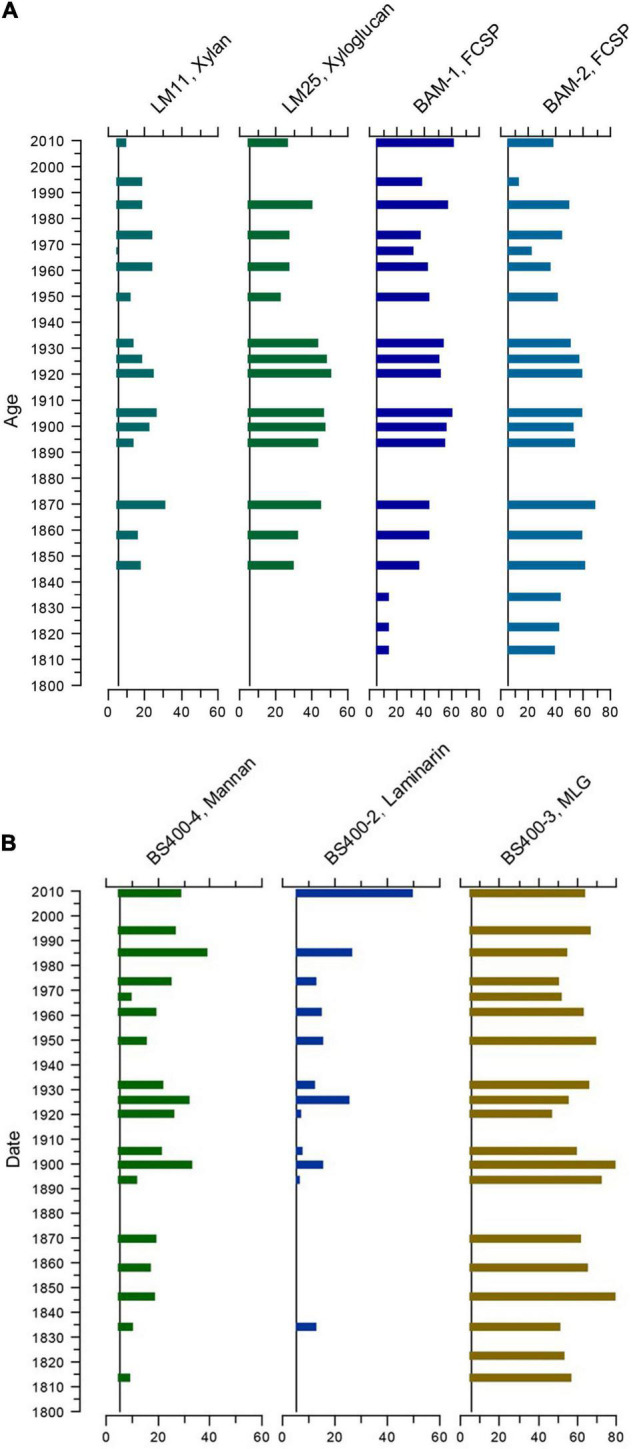
Fluctuations in selected epitopes by age. **(A)** More or less synchronous temporal fluctuations in the epitopes, detected by the mAbs: LM11 (1→4)-β-D-xylan/arabinoxylan; LM25 xyloglucan; BAM1 FCSP (un-sulfated epitope present in sulfated fucan) and BAM2 FCSP (sulfated epitope present in sulfated fucan). FCSP, fucose-containing sulfated polysaccharides. **(B)** Changes in relative presence of epitopes by age, detected by the mAbs: BS-400-4 (1→4)-β-D-mannan; BS-400-2 callose, laminarin (1→3)-β-D-glucan, and BS-400-3 (1→3)(1→4)-β-D-glucan. MLG, mixed-linkage glucan.

Due to the semiquantitative nature of the analysis, it is not possible to directly translate the strength of the signals to sedimentary concentrations and, therefore, not possible to make quantitative comparisons between the epitopes. However, within epitopes, signal strength indicates relative changes in sedimentary concentrations. Therefore, we plotted signal strength against the age of the samples for the epitopes with the strongest and/or most consistent signals. This revealed that some of the dominant epitopes fluctuated more or less synchronously (Figure 3A); however, not all showed the same pattern (Figure 3B). The possible implications of these patterns are discussed below.

## Discussion

### Methodology

To our knowledge, there are only a few other studies of the long-term preservation of carbohydrates in sediments. One study by Kaal et al. (2014) studied that lacustrine sediments and the sediment carbohydrates were detected by pyrolysis, another (Pancost and Boot, 2004) studied marine sediments, and the method in this study is also pyrolysis. Previous studies of carbohydrates in soil or sediment time series have mainly targeted monosaccharides and indicate that bacteria and cyanobacteria leave traces of fucose, ribose, mannose, and galactose (Moers and Larter, 1993). Other studies suggest that angiosperms leave xylose traces in parison with gymnosperms (Cowie and Hedges, 1984). It is possible to find other studies where they compare the ratio between simple sugars to distinguish between woody and non-woody materials (Cowie and Hedges, 1984).

This is the first time the antibody-based CoMPP method has been used to detect polysaccharides in marine sediments, and to our knowledge, this is the first time such a variety of polysaccharide epitopes have been detected in marine sediments. Compared with other traditional methods used to determine polysaccharides, CoMPP could become a strong asset for sediment analysis. Some of these methods can be less specific as it is the case of atomic C to N ratios (Bianchi and Canuel, 2011) or require a more laborious sample process as it is the case of sugar analysis in combination with gas chromatography-mass spectrometry (Pettolino et al., 2012).

### Variety and Preservation

We found a large variety of compounds preserved throughout much of the studied time period. The preservation of many epitopes throughout the core and the patterns of both increase and decrease over time indicate that the epitopes are responding to something other than degradation. A pure degradation pattern would be expected to take the shape of a more or less exponential decline. Although we would expect a pattern of resilience in the order of pectin > hemicelluloses > celluloses > lignin, due to the differences in degradability of these compounds (Albersheim et al., 2010), this is not reflected in the temporal epitope profiles. Therefore, we can conclude that it is very unlikely that the pattern we saw is purely a degradation pattern. We did not find the limit of preservation, as four of the epitopes showed signals continuously, all the way to the bottom of the core. One of the prerequisites for a geochemical proxy thus seems to be met, i.e., that the compounds can be preserved over time in the sediment.

### Provenance of the Epitopes

Many of the 30 epitopes for which we found signals in the sediment have been detected in previous studies in a variety of marine and freshwater land plants, seaweeds, and algae; a few have also been found in marine animals. The most dominant epitopes and their possible provenance are discussed below.

Among the polysaccharide epitopes detected in the samples, the most straightforward to mention is cellulose, which is detected by CBMs (i.e., CBM30 and CBM3a). In the Koljö Fjord samples, particularly CMB30 shows a consistently high signal throughout the core. All plants have cellulose in their cell walls and their content usually accounts for 35–50% of dry weight (Chen, 2014). Microalgae and phytoplankton (Vidal-Melgosa et al., 2021) also contain cellulose in their cell walls, and it is, therefore, not possible to conclude the extent to which the cellulose detected is of land plant or algal origin. Cellulose is difficult for many heterotrophic organisms to degrade (Voet and Voet, 2004) as it is made up of very stable microfibrils formed by a bundle of linear chains of glucose attached to each other by 1,4-β-glycosidic linkages. Plants contain large amounts of recalcitrant structural polysaccharides, while microalgae generally contain proportionally higher levels of protein. Due to proximity to land, it is likely that at least some of the cellulose detected was of land plant origin. However, due to the presence of cellulose in the cell walls of both macroalgae and microalgae, marine cellulose may also have contributed to the signals obtained.

Xylans, which are detected by the antibody LM11, are a structurally diverse group of polysaccharides commonly found in land plants (Vogel, 2008). They have also been found in smaller amounts in other members of the plant kingdom (Archaeplastida) such as green algae, comprising chlorophytes (Chlorophyta) and charophytes (Streptophyta), as well as red algae (Rhodophyta). However, there is no reference of xylans in the glaucophytes (Hsieh and Harris, 2019) or brown algae up to date (Michel et al., 2010; Hsieh and Harris, 2019). Thus, we believe that most of the signal produced by LM11 binding is likely to be from land plants.

(1→3)(1→4)-β-D-glucan (MLG), detected by the probe BS-400-3, is the epitope with the highest signal in the heat map and a consistently high signal throughout the core. MLG is restricted to certain taxonomic groups in the plant kingdom. While it is not present in dicotyledonous plants, it is a major glycan component of the cell walls of grasses and cereals of the Poaceae (Eder et al., 2008; Sørensen et al., 2008). It is also found in Equisetum (horsetails), lichens, fungi, and some bacteria. Furthermore, MLG is also present in some red and brown algae (Salmeán et al., 2017a and references therein). The high signals obtained with BS-400-3 may, therefore, be attributable to MLG from terrestrial or marine sources. The structural features of MLG, i.e., the amount and distribution of (1 → 3)-linked and (1 → 4)-linked glucans, varies according to the organism, and it may be possible to determine the source by undertaking structural analysis. However, this was beyond the scope of this study.

Callose (1→3)-β-D-glucan recognized by antibody BS-400-2 is a polysaccharide that occurs in land plants at certain stages of development, for example, during cell plate formation, and in response to some biotic stresses when it has a role in plant defense (Piršelová and Matušíková, 2013). Nevertheless, β-1,3-glucan is not generally abundant in land plants, and its occurrence is transient. (1→3)-β-D-glucan is also a structural feature of laminarin (sometimes known as laminaran)—an algal polysaccharide used as a storage glycan that is based on (1→3)-β-D-glucan with (1 →6)-β-D branches (Indergaard and Minsaas, 1991; Biersmith and Benner, 1998; Rioux et al., 2007), and laminarin can also be detected with BS-400-2.

A recent study (Becker et al., 2020) has shown that laminarin is a major molecule in the marine carbon cycle, and the moderate signals we saw in some samples are, therefore, most likely predominantly of algal origin.

Epitopes of fucose-containing sulfated polysaccharides (FCSP) also known as fucans or fucoidans are detected by BAM1 (an unsulfated epitope present in sulfated fucan), BAM2 (a sulfated epitope present in sulfated fucan), and BAM3 (a possibly sulfated epitope present in sulfated fucan) mAbs (Torode et al., 2015). Brown algal cell walls, particularly, contain these polysaccharides; however, some marine animals also contain FCSP in small amounts (Salmeán et al., 2017b). Several diatom species also contain FCSP (Vidal-Melgosa et al., 2021). The most likely provenance of these signals is, therefore, marine algae. The difference in detection of the three BAM probes is consistent with the results in seaweeds of Torode et al. (2015) and Vidal-Melgosa et al. (2021) and could give insights of specific provenance (e.g., order Fucales vs. Laminariales in the case of brown seaweeds).

We would like to remark that regardless of the presence of red seaweeds in the area; at the time when the analysis was performed, we lacked the right probes to determine the presence of sulfated galactans in the samples. However, these complex polysaccharides are known to be present in the cell walls of these aquatic organisms and could be found with the right analytical tools in the sediments. In a previous study, Salmeán et al. (2018) have used CBMs and SusD-like proteins as molecular probes; nevertheless, this is outside the scope of this sediment study. To our knowledge, there are still no molecular probes, which could be used with CoMMP to detect and distinguish the presence of sulfated galactans.

### Temporal Variation

**FIGURE 4:**
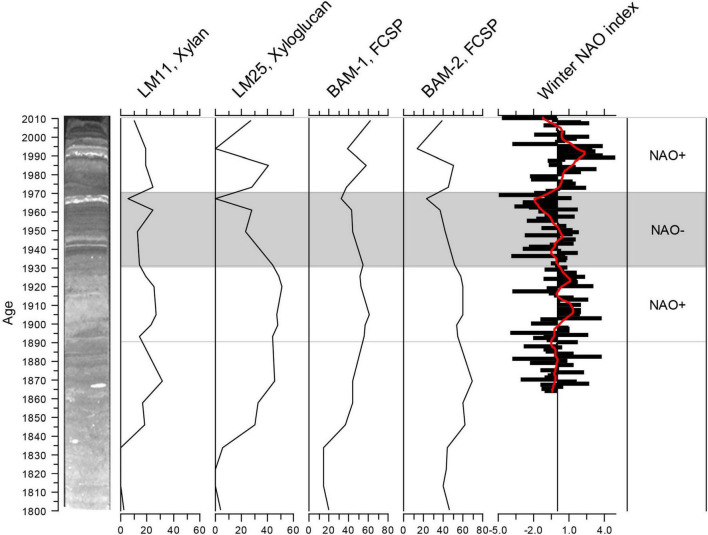
Fluctuations in the four epitopes from Figure 3A, compared with fluctuations in the winter North Atlantic Oscillation (NAO) index (refer to details in text).

There are clear temporal patterns in the intensity of the signals of many of the detected epitopes. Figure 3A shows almost synchronous fluctuations in four epitopes that all showed clear signals throughout the core. The general patterns of these fluctuations have some similarities with decadal trends in the NAO winter index (Hurrell, 2020). From ca. 1890 to ca. 1930 and from 1970 to 2000, the NAO is mainly positive (Figure 4). Positive NAO conditions have been linked to milder and wetter conditions in the Baltic area region (Schimanke et al., 2012), which would imply higher runoff of freshwater from land in these periods. This would fit a signal of the higher signal of terrestrially derived cell-wall epitopes in this period. The general decrease in cell-wall epitopes in the predominantly NAO-period (shaded area in Figure 4) is coherent with this interpretation. Apart from a decrease in 2003, the general trend at the top of the core is also increasing, which again approximately fits a return to positive NAO index ca. 1980. Another prerequisite for a geochemical proxy, therefore, also appears to be met, e.g., that they respond to environmental forcing. As the trends are not completely correlated and the data are so far from one core, our conclusion is that this method shows some promise for discovering potential new proxies for freshwater runoff but must be tested further.

Data on the temporal organic carbon content in a core from Koljö Fjord reported by Nordberg et al. (2001) show a fluctuating decrease from ca. 8% in ca. 1820 to ca. 5% from 1940 to 1960, then an increase to ca. 8% in ca. 1995, followed by a decrease to ca. 5% in ca. 2000. At the top of the core (younger than ca. 1950), this follows the pattern in the epitopes in Figures 3A, 4. However, the patterns diverge in the lower part of the core. In the samples older than ca. 1879, the signals in the epitopes in Figure 3A, and others, gradually decline with depth in the core. This may be a degradation signal.

### Future Perspectives

While there was a reduction in signal strength for some compounds with core depth, we did not, in this study, reach the limit for the preservation of these compounds, as at least four (CMB30, BS-400-3, BAM1, and BAM2) were present with strong signals to the bottom of the core at 67 cm (estimated to be ca. 200 years old). The maximal preservation limit for these compounds is, therefore, yet to be determined, e.g., by analyzing longer/older core material.

Given that this exploratory study is from one single location and a cold-temperate fjord setting, future study should also explore the longevity of these compounds in sediment records of different compositions and under different environmental settings.

As some of the compounds appear to be responding positively to the milder and wetter conditions of positive NAO index and as many of them likely are of terrestrial origin, they may have potential as paleoenvironmental proxies of terrestrial influence. As the method is simple and has high throughput, they could constitute a valuable contribution to geochemical proxies.

However, the response seen in this study should be confirmed. The preservation potential should be tested in other coastal (and perhaps also oceanic) locations. We saw from the pilot study that some of the epitopes were present at other sites, but this needs to be confirmed through down-core analyses. To test whether the potential proxies are in fact responding to terrestrial input, it would be relevant to analyze gradients from land, as they should, in this case, decline with distance, or in a river mouth (to test a potential relationship with salinity gradient).

To shed light on the role of these compounds in marine carbon cycling and their possible role in carbon sequestration, it would be relevant to look at gradients through the water column and into the sediment. Again, the high-throughput nature of this methodology is helpful.

At present, we cannot identify the epitopes with certainty, and we suggest elucidating this when we know which of them has the potential to be a robust, broadly applicable proxy and when it has been calibrated by further studies.

### Conclusion

We have shown that this method with its simple extraction and high-throughput analysis is applicable for analyzing epitopes of polysaccharides in marine sediment core samples. We have documented long-term preservation of a large variety of epitopes, in some cases, for more than 200 years. We further see fluctuations in relative abundance, apparently in some cases driven by environmental fluctuations.

## Data Availability Statement

The raw data supporting the conclusions of this article will be made available by the authors, without undue reservation.

## Author Contributions

AS, WW, and ME planned the study. AS and TA carried out the laboratory analyses. AS, ME, TA, and SR analyzed and interpreted the data. All authors contributed to writing the manuscript.

## Conflict of Interest

The authors declare that the research was conducted in the absence of any commercial or financial relationships that could be construed as a potential conflict of interest.

## Publisher’s Note

All claims expressed in this article are solely those of the authors and do not necessarily represent those of their affiliated organizations, or those of the publisher, the editors and the reviewers. Any product that may be evaluated in this article, or claim that may be made by its manufacturer, is not guaranteed or endorsed by the publisher.

## References

[B1] Albersheim P. Darvill A. Roberts K. Sederoff R. Staehelin A. (2010). *Plant Cell Walls.* New York, NY: Garland Science, 430.

[B2] Andersen T. J. (2017). “Some practical considerations regarding the application of ^210^pb and ^137^cs dating to estuarine sediments,” in *Applications of Paleoenvironmental Techniques in Estuarine Studies*, eds Weckström K. Saunders K. M. Gell P. A. Skilbeck C. G. (Berlin: Springer), 121–140. 10.1007/978-94-024-0990-1_6

[B3] Becker S. Tebben J. Coffinet S. Wiltshire K. Hvitfeldt Iversen M. Harder T. (2020). Laminarin is a major molecule in the marine carbon cycle. *Proc. Natl. Acad. Sci. U. S. A.* 117 6599–6607. 10.1073/pnas.1917001117 32170018 PMC7104365

[B4] Bianchi T. S. Canuel E. A. (2011). *Chemical Biomarkers in Aquatic Ecosystems Princeton.* Princeton, NJ: Princeton University Press 10.1515/9781400839100

[B5] Biersmith A. Benner R. (1998). Carbohydrates in phytoplankton and freshly produced dissolved organic matter. *Mar. Chem.* 63 131–144. 10.1016/s0304-4203(98)00057-7

[B6] Blake A. W. McCartney L. Flint J. E. Bolam D. N. Boraston A. B. Gilbert H. J. (2006). Understanding the biological rationale for the diversity of cellulose-directed carbohydrate-binding modules in prokaryotic enzymes. *J. Biol. Chem.* 281 29321–29329. 10.1074/jbc.M605903200 16844685

[B7] Boraston A. B. Notenboom V. Warren R. A. Kilburn D. G. Rose D. R. Davies G. (2003). Structure and ligand binding of carbohydrate-binding module CsCBM6-3 reveals similarities with fucose-specific lectins and “galactose-binding” domains. *J. Mol. Biol.* 327, 659–669. 10.1016/s0022-2836(03)00152-9 12634060

[B8] Chen H. (2014). “Chemical composition and structure of natural lignocellulose,” in *Biotechnology of Lignocellulose: Theory and Practice*, ed. Chen H. Z. (Dordrecht: Springer), 25–71. 10.1007/978-94-007-6898-7_2

[B9] Clausen M. H. Willats W. G. T. Knox J. P. (2003). Synthetic methyl hexagalacturonate hapten inhibitors of anti-homogalacturonan monoclonal antibodies LM7, JIM5 and JIM7. *Carbohydr. Res.* 338 1797–1800. 10.1016/s0008-6215(03)00272-6 12892947

[B10] Cossellu M. Nordberg N. (2010). Recent environmental changes and filamentous algal mats in shallow bays on the Swedish West coast – a result of climate change? *J. Sea Res*. 63 202–212. 10.1016/j.seares.2010.01.004

[B11] Cowie G. L. Hedges J. I. (1984). Carbohydrate sources in a coastal marine environment. *Geochim. Cosmochim. Acta* 48 2075–2087. 10.1016/0016-7037(84)90388-0

[B12] Cragg S. M. Friess D. A. Gillis L. G. Trevathan-Tackett S. M. Terrett O. M. Watts J. E. M. (2020). Vascular plants are globally significant contributors to marine carbon fluxes and sinks. *Ann. Rev. Mar. Sci.* 12 469–497. 10.1146/annurev-marine-010318-095333 31505131

[B13] Czjzek M. Bolam D. N. Mosbah A. Allouch J. Fontes C. M. Ferreira L. M. (2001). The location of the ligand-binding site of carbohydrate-binding modules that have evolved from a common sequence is not conserved. *J. Biol. Chem.* 76, 48580–48587. 10.1074/jbc.M109142200 11673472

[B14] Eder M. Tenhaken R. Driouich A. Lütz-Meindl U. (2008). Occurence and characterization of arabinogalactan-like proteins andhemicelluloses in *Micrasterias* (Streptophyta). *J. Phycol.* 44 1221–1234. 10.1111/j.1529-8817.2008.00576.x 27041719

[B15] Ellegaard M. Clarke A. L. Reuss N. Drew S. Weckström K. Juggins S. (2006). Multi-proxy evidence of long-term changes in ecosystem structure in a Danish marine estuary linked to increased nutrient loading. *Estuar. Coast. Shelf Sci.* 68 567–578. 10.1016/j.ecss.2006.03.013

[B16] Eriksson B. K. Johansson G. Snoeijs P. (2002). Long-term changes in the macroalgal vegetation of the inner Gullmar fjord, Swedish Skagerrak coast. *J. Phycol.* 38 284–296. 10.1046/j.1529-8817.2002.00170.x

[B17] Fernandes A. C. Fontes C. M. Gilbert H. J. Hazlewood G. P. Fernandes T. H. Ferreira L. M. (1999). Homologous xylanases from Clostridium thermocellum: evidence for bi-functional activity, synergism between xylanase catalytic modules and the presence of xylan-binding domains in enzyme complexes. *Biochem. J.* 342(Pt 1), 105–110. 10432306 PMC1220442

[B18] Field C. B. Behrenfeld M. J. Randerson J. T. Falkowski P. (1998). Primary production of the biosphere: integrating terrestrial and oceanic components. *Science* 281 237–240. 10.1126/science.281.5374.237 9657713

[B19] Gontikaki E. Thornton B. Cornulie T. Witte U. (2015). Occurrence of priming in the degradation of lignocellulose in marine sediments. *PLoS One* 10:e0143917. 10.1371/journal.pone.0143917 26633175 PMC4669084

[B20] Gustafsson M. Nordberg K. (1999). Benthic foraminifera and their response to hydrography, periodic hypoxic conditions and primary production in the Koljo fjord on the Swedish west coast. *J. Sea Res.* 41 163–178. 10.1016/s1385-1101(99)00002-7

[B21] Harland R. Nordberg K. Filipsson H. L. (2004). A high-resolution dinoflagellate cyst record from latest Holocene sediments in Koljö Fjord. *Sweden. Rev. Palaeobot. Palynol.* 128 119–141. 10.1016/s0034-6667(03)00116-7

[B22] Hedges J. I. Keil R. G. Benner R. (1997). What happens to terrestrial organic matter in the ocean? *Org. Geochem.* 27 195–212. 10.1016/j.envpol.2016.07.057 27481644

[B23] Henshaw J. L. Bolam D. N. Pires V. M. Czjzek M. Henrissat B. Ferreira L. M. (2004). The family 6 carbohydrate binding module CmCBM6-2 contains two ligand-binding sites with distinct specificities. *J. Biol. Chem.* 279, 21552–21559. 10.1074/jbc.M401620200 15004011

[B24] Hernandez-Gomez M. C. Rydahl M. G. Rogowski A. Morland C. Cartmell A. Crouch L. (2015). Recognition of xyloglucan by the crystalline cellulose-binding site of a family 3a carbohydrate-binding module. *FEBS Lett.* 589 2297–2303. 10.1016/j.febslet.2015.07.009 26193423 PMC5877785

[B25] Hervé C. Siméon A. Jam M. Cassin A. Johnson K. L. Salmeán A. A. (2016). Arabinogalactan proteins have deep roots in eukaryotes: identification of genes and epitopes in brown algae and their role in *Fucus serratus* embryo development. *New Phytol.* 209 1428–1441. 10.1111/nph.13786 26667994

[B26] Hsieh Y. S. Y. Harris P. J. (2019). Xylans of red and green algae: What is known about their structures and how they are synthesised? *Polymers* 11:354. 10.3390/polym11020354 30960338 PMC6419167

[B27] Hurrell J. (2020). *The Climate Data Guide: Hurrell North Atlantic Oscillation (NAO) Index (station-based).* Boulder, CO: National Center for Atmospheric Research Staff.

[B28] Indergaard M. Minsaas J. (1991). “Animal and human nutrition,” in *Seaweed Resources in Europe: Uses and Potential*, eds Guiry M. D. Blunden G. (Chichester: John Wiley & Sons), 21–64.

[B29] Jia G. Dungait J. A. J. Bingham E. M. Valiranta M. Korhola A. Evershed R. P. (2008). Neutral monosaccharides as biomarker proxies for bog-forming plants for application to palaeovegetation reconstruction in ombrotrophic peat deposits. *Org. Geochem.* 39 1790–1799. 10.1016/j.orggeochem.2008.07.002

[B30] Jones L. Seymour G. B. Knox J. P. (1997). Localization of pectic galactan in tomato cell walls using a monoclonal antibody specific to (1[->]4)-[beta]-D-galactan. *Plant Physiol.* 113 1405–1412. 10.1104/pp.113.4.1405 12223681 PMC158264

[B31] Kaal J. Schellekens J. Nierop K. G. J. Cotizas A. M. Muller J. (2014). Contribution of organic matter molecular proxies to interpretation of the last 55ka of the Lynch’s Crater record (NE Australia). *Palaeogeogr. Palaeoclimatol. Palaeoecol.* 414 20–31.

[B32] Kaiser M. (2011). *Marine Ecology – Processes, Systems and Impacts.* Oxford: Oxford University Press.

[B33] Knox J. P. Linstead P. J. Peart J. Cooper C. Roberts K. (1991). Developmentally regulated epitopes of cell surface arabinogalactan proteins and their relation to root tissue pattern formation. *Plant J.* 1 317–326. 10.1046/j.1365-313X.1991.t01-9-00999.x 21736649

[B34] Lee K. J. D. Sakata Y. Mau S.-L. Pettolino F. Bacic A. Quatrano R. S. (2005). Arabinogalactan proteins are required for apical cell extension in the moss *Physcomitrella patens*. *Plant Cell* 17 3051–3065. 10.1105/tpc.105.034413 16199618 PMC1276029

[B35] Marcus S. E. Blake A. W. Benians T. A. S. Lee K. J. D. Poyser C. Donaldson L. (2010). Restricted access of proteins to mannan polysaccharides in intact plant cell walls. *Plant J.* 64 191–203. 10.1111/j.1365-313X.2010.04319.x 20659281

[B36] Marcus S. E. Verhertbruggen Y. Hervé C. Ordaz-Ortiz J. J. Farkas V. Petersen H. L. (2008). Pectic homogalacturonan masks abundant sets of xyloglucan epitopes in plant cell walls. *BMC Plant Biol.* 8:60. 10.1186/1471-2229-8-60 18498625 PMC2409341

[B37] McCabe P. F. Valentine T. A. Forsberg L. S. Pennell R. I. (1997). Soluble signals from cells identified at the cell wall establish a developmental pathway in carrot. *Plant Cell* 9 2225–2241. 10.1105/tpc.9.12.2225 12237357 PMC157070

[B38] McCartney L. Marcus S. E. Knox J. P. (2005). Monoclonal antibodies to plant cell wall Xylans and Arabinoxylans. *J. Histochem. Cytochem*. 53 543–546. 10.1369/jhc.4B6578.2005 15805428

[B39] McQuoid M. R. Nordberg K. (2003). Environmental influence on the diatom and silicoflagellate assemblages in koljö fjord (Sweden) over the last two centuries. *Estuaries* 26 927–937. 10.1007/bf02803351

[B40] Meikle P. J. Bonig I. Hoogenraad N. J. Clarke A. E. Stone B. A. (1991). The location of (1–>3)-beta-glucans in the walls of pollen tubes of *Nicotiana alata* using a (1–>3)-beta-glucan-specific monoclonal antibody. *Planta* 185 1–8. 10.1007/BF00194507 24186272

[B41] Meikle P. J. Hoogenraad N. J. Bonig I. Clarke A. E. Stone B. A. (1994). A (1→ 3, 1→ 4)-β-glucan specific monoclonal antibody and its use in the quantitation and immunocytochemical location of (1→ 3, 1→ 4)-β-glucans. *Plant J.* 5 1–9. 10.1046/j.1365-313x.1994.5010001.x 8130794

[B42] Michel G. Tonon T. Scornetm D. Cock J. M. Kloareg B. (2010). The cell wall polysaccharide metabolism of the brown alga *Ectocarpus siliculosus*. Insights into the evolution of extracellular matrix polysaccharides in Eukaryotes. *New Phytol.* 188 82–97. 10.1111/j.1469-8137.2010.03374.x 20618907

[B43] Moers M. E. C. Larter S. R. (1993). Neutral monosaccharides from a hypersaline tropical environment: applications to the characterization of modern and ancient ecosystems. *Geochim. Cosmochim. Acta* 57 3063–3071. 10.1016/0016-7037(93)90293-6

[B44] Moller I. Marcus S. E. Haeger A. Verhertbruggen Y. Verhoef R. Schols H. (2008). High-throughput screening of monoclonal antibodies against plant cell wall glycans by hierarchical clustering of their carbohydrate microarray binding profiles. *Glycoconj J.* 25 37–48. 10.1007/s10719-007-9059-7 17629746 PMC2234451

[B45] Moller I. Sorensen I. Bernal A. J. Blaukopf C. Lee K. Obro J. (2007). High-throughput mapping of cell-wall polymers within and between plants using novel microarrays. *Plant J.* 50, 1118–1128. 10.1111/j.1365-313X.2007.03114.x 17565618

[B46] Najmudin S. Guerreiro C. I. Carvalho A. L. Prates J. A. Correia M. A. Alves V. D. (2006). Xyloglucan is recognized by carbohydrate-binding modules that interact with beta-glucan chains. *J. Biol. Chem*. 281 8815–8828. 10.1074/jbc.M510559200 16314409

[B47] Nordberg K. Filipsson H. L. Gustafsson M. Harland R. Roos P. (2001). Climate, hydrographic variations and marine benthic hypoxia in Koljö Fjord. *Sweden. J. Sea Res.* 46 187–200. 10.1016/s1385-1101(01)00084-3

[B48] Pancost R. D. Boot C. S. (2004). The palaeoclimatic utility of terrestrial biomarkers in marine sediments. *Mar. Chem.* 92 239–261. 10.1016/j.marchem.2004.06.029

[B49] Pedersen H. L. Fangel J. U. McCleary B. Ruzanski C. Rydahl M. G. Ralet M. C. (2012). Versatile high resolution oligosaccharide microarrays for plant glycobiology and cell wall research. *J. Biol. Chem.* 287 39429–39438. 10.1074/jbc.M112.396598 22988248 PMC3501085

[B50] Pennell R. I. Knox J. P. Scofield G. N. Selvendran R. R. Roberts K. (1989). A family of abundant plasma membrane-associated glycoproteins related to the arabinogalactan proteins is unique to flowering plants. *J. Cell Biol*. 108 1967–1977. 10.1083/jcb.108.5.1967 2469683 PMC2115552

[B51] Pettolino F. Walsh C. Fincher G. Bacic A. (2012). Determining the polysaccharide composition of plant cell walls. *Nat. Protoc.* 7 1590–1607. 10.1038/nprot.2012.081 22864200

[B52] Pettolino F. A. Hoogenraad N. J. Ferguson C. Bacic A. Johnson E. Stone B. A. (2001). A (1-4)-beta-mannan-specific monoclonal antibody and its use in the immunocytochemical location of galactomannans. *Planta* 214 235–242. 10.1007/s004250100606 11800387

[B53] Pires V. M. Henshaw J. L. Prates J. A. Bolam D. N. Ferreira L. M. Fontes C. M. (2004). The crystal structure of the family 6 carbohydrate binding module from *Cellvibrio mixtus* endoglucanase 5a in complex with oligosaccharides reveals two distinct binding sites with different ligand specificities. *J. Biol. Chem.* 279, 21560–21568. 10.1074/jbc.M401599200 15010454

[B54] Piršelová B. Matušíková I. (2013). Callose: the plant cell wall polysaccharide with multiple biological functions. *Acta Physiol. Plant* 35 635–644. 10.1007/s11738-012-1103-y

[B55] Ralet M.-C. Tranquet O. Poulain D. Moïse A. Guillon F. (2010). Monoclonal antibodies to rhamnogalacturonan I backbone. *Planta* 231 1373–1383. 10.1007/s00425-010-1116-y 20309579

[B56] Reuss N. Conley D. J. Bianchi T. S. (2005). Preservation conditions and the use of sediment pigments as a tool for recent ecological reconstruction in four Northern European estuaries. *Mar. Chem.* 95 283–302. 10.1016/j.marchem.2004.10.002

[B57] Ribeiro S. Berje T. Lundholm N. Andersen T. J. Abrantes F. Ellegaard M. (2011). Phytoplankton growth after a century of dormancy illuminates past resilience to catastrophic darkness. *Nat. Commun.* 2:311. 10.1038/ncomms1314 21587228 PMC3113231

[B58] Rioux L.-E. Turgeon S. L. Beaulieu M. (2007). Characterization of polysaccharides extracted from brown seaweeds. *Carbohydr. Polym.* 69 530–537. 10.1016/j.carbpol.2007.01.009

[B59] Salmeán A. A. Duffieux D. Harholt J. Fen Qin F. Gurvan Michel G. Mirjam Czjzek M. (2017a). Insoluble (1 → 3), (1 → 4)-β-D-glucan is a component of cell walls in brown algae (Phaeophyceae) and is masked by alginates in tissues. *Sci. Rep.* 7:2880. 10.1038/s41598-017-03081-5 28588313 PMC5460208

[B60] Salmeán A. A. Hervé C. Jørgensen B. Willats W. G. T. Mravec J. (2017b). Microarray glycan profiling reveals algal fucoidan epitopes in diverse marine metazoans. *Front. Mar. Sci.* 4:293. 10.3389/fmars.2017.00293

[B61] Salmeán A. A. Guillouzo A. Duffieux D. Jam M. Matard-Mann M. Larocque R. (2018). Double blind microarray-based polysaccharide profiling enables parallel identification of uncharacterized polysaccharides and carbohydrate-binding proteins with unknown specificities. *Sci. Rep.* 8:2500. 10.1038/s41598-018-20605-9 29410423 PMC5802718

[B62] Schimanke S. Meier H. E. M. Kjellström E. Strandberg G. Hordoir R. (2012). The climate in the Baltic Sea region during the last millennium simulated with a regional climate model. *Clim. Past* 8 1419–1433.

[B63] Smallwood M. Beven A. Donovan N. Neill S. J. Peart J. Roberts K. (1994). Localization of cell wall proteins in relation to the developmental anatomy of the carrot root apex. *Plant J.* 5 237–246.

[B64] Smittenberg R. H. Pancost R. D. Hopmans E. C. Paetzel M. Sinninghe Damsté J. S. (2004). A 400-year record of environmental change in a euxinic fjord as revealed by the sedimentary biomarker record. *Palaeogeogr. Palaeoclimatol. Palaeoecol.* 202 331–351. 10.1016/s0031-0182(03)00642-4

[B65] Sørensen I. Pettolino F. A. Wilson S. M. Doblin M. S. Johansen B. Bacic A. (2008). Mixed-linkage (1 → 3), (1 → 4)-ß-D-glucan is not unique to the Poales and is an abundant component of *Equisetum arvense* cell walls. *Plant J.* 54 510–521. 10.1111/j.1365-313X.2008.03453.x 18284587

[B66] Spohn M. Giani L. (2012). Carbohydrates, carbon and nitrogen in soils of a marine and a brackish marsh as influenced by inundation frequency. *Estuar. Coast. Shelf Sci*. 107 89–91. 10.1016/j.ecss.2012.05.006

[B67] Torode T. A. Marcus S. E. Jam M. Tonon T. Blackburn R. S. Hervé C. (2015). Monoclonal antibodies directed to fucoidan preparations from brown algae. *PLoS One* 10:e0118366. 10.1371/journal.pone.0118366 25692870 PMC4333822

[B68] van Loon H. Rogers J. C. (1978). The seesaw in winter temperatures between Greenland and Northern Europe. Part I: general description. *Mon. Weather Rev.* 106 296–310. 10.1175/1520-0493(1978)106<0296:tsiwtb>2.0.co;2

[B69] Verhertbruggen Y. Marcus S. E. Haeger A. Verhoef R. Schols H. A. McCleary B. V. (2009). Developmental complexity of arabinan polysaccharides and their processing in plant cell walls. *Plant J.* 59 413–425. 10.1111/j.1365-313X.2009.03876.x 19392693

[B70] Vidal-Melgosa S. Sichert A. Francis T. B. Bartosik D. Niggemann J. Wichels A. (2021). Diatom fucan polysaccharide precipitates carbon during algal blooms. *Nat. Commun.* 12:1150. 10.1038/s41467-021-21009-6 33608542 PMC7896085

[B71] Voet D. Voet J. G. (2004). in *Biochemistry / Donald Voet*, 3rd Edn, ed. Voet J. G. (Hoboken, NJ: J. Wiley & Sons).

[B72] Vogel J. (2008). Unique aspects of the grass cell wall. *Curr. Opin. Plant Biol.* 11 301–307. 10.1016/j.pbi.2008.03.002 18434239

[B73] Willats W. G. T. Marcus S. E. Knox J. P. (1998). Generation of a monoclonal antibody specific to (1í5)-alpha-L-arabinan. *Carbohydr. Res*. 308 149–152. 10.1016/s0008-6215(98)00070-6 9675359

[B74] Willats W. G. T. McCartney L. Steele-King C. G. Marcus S. E. Mort A. Huisman M. (2004). A xylogalacturonan epitope is specifically associated with plant cell detachment. *Planta* 218 673–681. 10.1007/s00425-003-1147-8 14618325

[B75] Willats W. G. T. Orfila C. Limberg G. Buchholt H. C. van Alebeek G. J. Voragen A. G. (2001). Modulation of the degree and pattern of methyl-esterification of pectic homogalacturonan in plant cell walls: implications for pectin methyl esterase action, matrix properties, and cell adhesion. *J. Biol. Chem*. 276 19404–19413. 10.1074/jbc.M011242200 11278866

[B76] Youssef D. H. El-Said G. F. Shobier A. H. (2014). Distribution of total carbohydrates in surface sediments of the Egyptian Mediterranean coast, in relation to some inorganic factors. *Arabian J. Chem.* 7 823–832. 10.1016/j.arabjc.2012.12.030

